# Factors influencing utilization of skilled birth attendant during childbirth in the Southern highlands, Tanzania: a multilevel analysis

**DOI:** 10.1186/s12884-020-03110-8

**Published:** 2020-07-25

**Authors:** Damian J. Damian, Judges Y. Tibelerwa, Beatrice John, Rune Philemon, Michael J. Mahande, Sia E. Msuya

**Affiliations:** 1 Department of Epidemiology and Biostatistics, Institute of Public Health, KCMUCo, Moshi, Tanzania; 2 Community Health Department, Kilimanjaro Christian Medical Centre (KCMC), Moshi, Tanzania; 3 Department of Community Health, Institute of Public Health, Kilimanjaro Christian Medical University College (KCMUCo), Moshi, Tanzania; 4 Department of Paediatrics & Child Health, KCMUCo & Kilimanjaro Christian Medical Centre (KCMC), Moshi, Tanzania

**Keywords:** Utilization, Skilled births attendants, SBA, Factors, Predictors, Southern highlands, Tanzania

## Abstract

**Background:**

Use of skilled health provider (SBA) during and after childbirth has been reported to reduce maternal and newborn deaths; and is one of the key indicators monitored in Sustainable Development Goals (SDGs). Progress, levels and factors influencing utilization of SBA differ within and between countries. In Tanzania, SBA coverage stands at 64% while the national target is 80%; with wide variability between regions (42–96%). This study aimed at determining factors associated with utilization of skilled births providers during childbirth in Mbeya Region, Southern highlands, Tanzania.

**Methods:**

This was a cross-sectional analytical study conducted in December 2015 to January 2016, in Mbeya Region. A total of 2844 women of reproductive age were enrolled, but only 1777 women who reported a live birth 5 years prior to the survey were included in this analysis. Multilevel logistic regression analyses were used to determine independent factors influencing utilization of SBA during childbirth. Random effects logistic model was used to assess the variability between clusters on the odds of using skilled birth attendants during delivery.

**Results:**

In this setting, 81% of the women reported utilization of skilled births attendants during childbirth. ANC visits four times or more (aOR = 1.63–95% CI = 1.26, 2.10; *p* < 0.001) and having secondary education or higher (aOR = 2.16; 95% CI = 1.19–3.90; *p* = 0.011) were associated with increased SBA use during childbirth whereas having two (aOR = 0.51; 95% CI: 0.33–0.79; *p* = 0.003) or three children (aOR = 0.37; 95% CI: 0.27–0.58; *p* < 0.001) relative to one child, 30 to 60 min walking distance to the health facility (aOR = 0.66; 95% CI: 0.48–0.92; *p* = 0.012) and more than 1 h walking distance to the health facility (aOR = 0.43; 95% CI: 0.32–0.57; *p* < 0.001) compared to < 30 min; were associated with decreased SBA use during childbirth.

**Conclusion:**

The proportion of births attended by skilled births attendants was high, but 19% of the women are still left behind. Concentrated efforts to improve utilization of SBA should be targeted to women with low education, with higher number of children, and with low frequency of ANC attendance. Furthermore, community and facility interventions addressing transport for pregnant women are needed. Qualitative study to explore the barriers of SBA use among the 19% who are not using skilled assistance during childbirth is needed.

## Plain English summary

Assistance by a health professional such as midwife, doctor or nurses who has been educated and trained to provide essential and emergency obstetric care to women and their newborns during pregnancy, childbirth and the immediate postnatal period is known to reduce maternal and newborn deaths. In this study, the investigators interviewed and analysed data for 1777 women aged 15 to 49 years who reported a live birth in the past 5 years prior to the study in Mbeya region. The aim was to assess how many women were assisted by trained personnel during childbirth and associated reasons. Eight out of ten women reported to be assisted by trained health professionals during childbirth. Utilisation was found to be improved by the level of education of a woman i.e. at least secondary education; short distance to the health facility; having fewer children and attending four or more ANC visits. In conclusion, still a substantial proportion of women (19%) are left behind in using this life saving intervention.

## Background

Skilled and competent care provided to women before, during and after childbirth by skilled health personnel is critical in saving women and newborn lives [1, 2]. Skilled birth attendant (SBA) as defined by WHO, is an accredited health professional such as midwife, doctor or nurses who has been educated and trained to proficiency in the skills needed to manage women during normal (uncomplicated) childbirth and the immediate postnatal period as well as in the identification, management or referral of complications in women and newborns [3, 4]. Globally, there were approximately 295,000 maternal deaths in 2017 and 2.5 million neonatal deaths in 2018, nearly 94% of these occur in low resource settings [1, 5]. Studies have however shown that, 66% of maternal deaths and 43% of neonatal deaths can be prevented in births that occur with assistance of SBA [1, 6]. That’s why increasing births assisted by skilled health personnel to 90% is one of the key indicators in the Sustainable Development Goal (SDG) goal number 3.1 [7].

SBA matters in ending preventable maternal deaths because of evidence from historical cohorts [2, 8, 9]. Maternal mortality can be successfully reduced in developing countries with investment in enabling policies, enabling environment, expansion of deliveries by skilled personnel in rural and underserved areas, and remove of financial barriers [8]. Sweden reduced its maternal mortality ratio (MMR) from 600 to 230 within 25 years when deliveries by trained professional midwives became widely available [9]. World Bank in its 2003 publication showed different levels of MMR depending on SBA coverage in countries with similar GDP. Sri Lanka and Malaysia with SBA coverage of more than 90% had MMR < 100 per 100,000 live births while other countries with SBA coverage of 40–50% had MMR ranging from 500 to 800 per 100,000 live births [8].

The primary cause of maternal deaths is direct obstetric causes that accounts for almost 75% of all maternal deaths. Haemorrhage (25%), pre-eclampsia and eclampsia (16%), infections (10%), unsafe abortions (10%) and other direct causes such as ectopic pregnancy, embolism and anaesthesia (9%) are the leading causes of these deaths [1, 2, 10]. Most of these deaths occur during childbirth and within the first 24 h after delivery. Critical issue is that these causes of maternal deaths can be managed properly at health facilities that can offer emergency obstetric care and have skilled health personnel [2]. It is expected that SBA can detect and manage the obstetric complications in timely manner or stabilize and refer the women and newborns with complications depending on the level of care [2, 11, 12]. Thus, majority of maternal and newborn deaths are preventable with availability of SBA.

The key problem is that coverage of births attended by skilled health personnel is still low in most of sub Saharan Africa (SSA) countries, while it’s the region accounting for 66% of global maternal deaths [1, 13]. Globally, the proportion of births attended by skilled health personnel has increased from 59% in 1990 to 71% in 2015, and up to 80% in 2017 [1, 4, 13]. In SSA only 59% of births were assisted by skilled health personnel in 2017 compared to 90–95% in South America, and 99% in high income countries [13]. This proportion is below the global goal of having SBA coverage of 90% for all births [2, 7].

Tanzania like other SSA countries has very high maternal mortality ratio (MMR) of 524 per 100,000 live births according to WHO estimates of 2017, and an SBA coverage below the international and national targets [1, 14]. Tanzania national target is to have 80% of births attended by skilled health personnel by 2020 [14, 15]. SBA coverage increased from 46% in 2004, to 51% in 2010 and 64% in 2016, which is still far from the country and international targets of SBA coverage [4, 15, 16]. Within the country, SBA coverage varies widely across regions, ranging from 42 to 96% [15]. Out of the 31 regions in the country, 3 regions have SBA coverage of ≥90%, 5 regions have SBA coverage between 80 and 87% and the rest have SBA coverage below the national target of 80% [14, 15].

Wealth, education level, place of residence and frequency of antenatal care visits have shown to influence use of skilled health personnel during and immediate after childbirth in many settings [15–28]. In Tanzania, Demographic and Health Survey gives level of SBA coverage and trends over time [15, 16]. It does not assess regional factors influencing the use of skilled health personnel during childbirth, information which important for local planning given the regional variations in SBA use. The need to study who is left behind in regions with lower SBA coverage is important in Tanzania [15]. This study aimed to determine the level and factors influencing utilization of skilled birth attendants during childbirth in Mbeya region, in South West of Tanzania. At the time of planning this study, Mbeya region had 65% of births assisted by SBA compared to neighbouring regions of Njombe and Iringa which had SBA coverage of 86 and 93% respectively [15, 16].

## Methods

### Study design and setting

This was a cross-sectional analytical study design which was conducted in Mbeya Region, from December 2015 to January 2016. Mbeya Region is situated in the Southern Highlands of Tanzania. It consists of 11 districts including Mbeya City, Mbeya District Council, Kyela, Rungwe, Mbarali, Ileje, Mbozi, Chunya, Tunduma, Busokelo and Momba. According to the 2012 census, Mbeya region had a total population of 2,707,410 (1,297,738 male and 1,409,672 female), and 663,143 of the women were of reproductive age. The region has 415 health facilities including hospitals, health centre and dispensaries [29].

According to 2010 TDHS report, in Mbeya region, 93% of the women of reproductive age attended ANC at least once, 43% of the women with live births in the past 5 years preceding the study were utilizing skilled births attendants during delivery and 5.3% of the women received first postnatal health check-up within 2 days of delivery [16].

### Population and enrolment procedures

The population for this study included women of reproductive age (15–49 years) who were residents in the selected households of Mbeya region. The analysis for SBA use during childbirth included women of reproductive age with a live birth within the past 5 years prior to the survey.

The sample size was estimated using multiple indicator cluster survey formula developed by the UN [30].

$$ \mathrm{N}=\frac{\left[3.84\ \left(\mathrm{r}\right)\ \left(1\hbox{-} \mathrm{r}\right)\ \left(\mathrm{f}\right)\ (1.1)\right]}{\left[{\left(0.12\mathrm{r}\right)}^2\left(\mathrm{p}\right)\ \left({\mathrm{n}}_{\mathrm{h}}\right)\right]} $$

Where

- **▪ n** is the required sample size, expressed as number of households, for the key indicator (see following section on determining the key indicator)
- **▪ 3.84** is a factor to achieve the 95% level of confidence
- **▪ r** is the predicted or anticipated prevalence for the indicator being estimated i.e. SBA coverage in Mbeya Region – 43%
- **▪ 1.1** is the factor necessary to raise the sample size by 10% for non-response
- **▪ f** is the shortened symbol for *deff* (i.e. design effect*)* = 2.0
- **▪ 0.12r** is the margin of error to be tolerated at the 95% level of confidence, defined as 12% of r (12% thus represents the relative sampling error of r)
- **▪ p** is the proportion of the total population upon which the indicator, r, is based (i.e. women of reproductive age: 25% [29]
- **▪ n**_**h**_ is the average household size (i.e. = 4 by Census Report, 2013)
- **▪** Cluster size was be set at 30

Using the formula above, the minimum sample size of the women of reproductive age (WRA) (N) obtained was 3595. Effective number of households required was 3595/0.9 = 3995 households. Thus, the minimum sample size of WRA required was 3995. The study needed 133 enumeration areas (effective number of households/cluster size of 30; 3995/30). Allocation of enumeration areas to the districts was guided by the proportion of women per each district. However, during the study period, there was a new district established. With this reason, some EAs were added to our sample such that, a total of 99 EAs were sampled. Information on EAs was obtained from the National Bureau of Statistics (NBS) of Tanzania, the implementing agency for the Tanzania and Demographic Health Surveys.

A multistage sampling technique was used to obtain WRA in all districts of Mbeya region. The multistage sampling techniques involved three stages. First stage involved simple random selection of enumeration areas in each of the eleven districts which was done by an expert from NBS. The second stage involved a systematic sampling of households from each of the selected enumeration area. The third stage was at the household level whereby all women of reproductive age were invited to participate in the survey. In total, 2844 women of reproductive age were enrolled in this study.

### Data collection methods and procedures

Data were collected during face-to-face interviews by trained research assistants. The interviews were conducted at either participant’s home or at the ward/village office depending on the preference of the participant. The questionnaires were administered in Swahili language.

The questionnaire used was developed for this study and had both closed and open-ended questions (Supplementary file 1). The questionnaire had a total of five sections. The first section collected information on socio-demographic and economic characteristics of the participants (i.e. age, education, marital status, employment, partner’s age and education, woman’s income, family income and housing). The second part included information on the reproductive and maternal health history (parity, number of living children, history of abortion/miscarriages, history of stillbirths, information on ANC use in last pregnancy, information on place of delivery, use of skilled birth attendants during childbirth and during postnatal period. Knowledge of danger signs during pregnancy, labour and childbirths and after delivery, information on birth preparedness and complication readiness (BPCR) plan in the last pregnancy, as well as information about distance and cost to the facilities was covered in the third section. The fourth section had questions on satisfaction and future intention to use the services at health facilities, while section 5 covered information on contraceptive use and breastfeeding practices after delivery. All the questions used in the questionnaire for section 1–3 and 5 were questions from the Tanzania Demographic and Health Survey questionnaire.

A survey team had a total of 40 people: 35 research assistants with background in medicine or public health and 5 field supervisors. All research assistants underwent 3 days intense training on the study, data collection tools and data collection procedures before field work commenced. The questionnaire was first pre-tested with ten women at in Moshi municipal where the research team was based. Thereafter, all necessary changes to the questions were made. The team pre-tested the questionnaire for the second time in Mbeya Region with five women who were attending a routine care in health facilities (Mbeya City). Few questions were thereafter adjusted following feedbacks before the actual data collection. On average, the interviews lasted for about 45 min.

### Variables and definitions

#### SBA use

A woman was categorized that she has used skilled birth attendant during childbirth if she was assisted by any of the following provider in her last pregnancy; a doctor, an assistant medical officer (AMO), clinical officer, an assistant clinical officer, nurse/midwives, and maternal and child health (MCH) aide [2].

#### Knowledge of danger signs

Women were asked to mention the danger signs of obstetric emergency that they know during pregnancy, childbirth and immediately after childbirth. Key danger signs during pregnancy include vaginal bleeding, symptoms and signs of severe pre-eclampsia (swollen face or hands, headache and blurred vision), fits/ convulsions, cessation of fetal movement and high fever. Key danger signs during childbirth include severe bleeding, prolonged labour (> 12 h), fits/ convulsions and retained placenta. Severe vaginal bleeding, fits/convulsions, high fever, and foul-smelling discharge are among key danger signs immediately after delivery. The women were considered knowledgeable (good knowledge) of danger signs of obstetric emergencies during pregnancy, delivery and after delivery if they mentioned three or more danger signs at each period. Those who mentioned less than three were categorized as having poor knowledge.

#### Birth preparedness and complication readiness plan

BPCR plan has six components i.e. a woman has to prepare or choose health facility of delivery, prepare a provider who will help with delivery, prepare money for transport when labour start, prepare transport in case of emergency, prepare money in case operation needed and prepare potential blood donors if needed. A woman was well prepared if she prepared for 3 or more components.

A woman was classified as having birth preparedness and complication readiness plan (well prepared) if she reported that she had prepared three or more of the six components of birth prepared and complication readiness plan in the last pregnancy. Otherwise she was recorded not being well prepared [15, 18–20].

#### Other variables

Socio- demographic variables that were included in the analysis were; age (15–24, 25–34, 35–49), education level (none, primary, secondary and above), marital status (married/cohabiting, single, divorced/widowed/separated), residence (urban vs. rural). Socio-economic characteristics: income of the participants (< 30 USD per month vs. ≥ 30), women employment status (employed vs. not employed) and types of accommodation (living in own house, rented or living with relatives). Partners age (< 24, 25–34, 35–44, 45–54) and partners education level were also assessed.

Distance to the nearest health facility with delivery services while walking (≤ 30 min, 31–60, > 60 min): fare used from home to health facility with delivery services (< 2000 TZS, 2000–5000, > 5000 TZS) and mode of transport used during last delivery (walking, motorcycle and car) were other variables assessed.

Reproductive and maternal health: number of pregnancies, number of children, history of stillbirths and neonatal death, attendance for antenatal care at least once during pregnancy, gestation age at 1st attendance or visit and frequency of ANC visit in last pregnancy, place of delivery and assistance during delivery were other variables assessed.

Reasons for home delivery: This was an open-ended question aimed at exploring detailed information for home delivery. Women were asked to mention the reasons for home delivery whereby multiple responses were possible. These reasons were thereafter coded, summarized into key thematic areas and presented in the manuscript.

### Statistical analysis

Data were analysed using Stata Version 15 (Stata Corporation, College station. Tx, USA). Categorical variables were summarized using frequency and percentage while numerical variables were summarized using mean and Standard Deviation (SD). Factors influencing the utilization of SBA during delivery were estimated in a multilevel regression analysis. Random effects logistic regression model was used to assess the variability between clusters on the odds of using skilled birth attendant during delivery. In our data, district was defined as a cluster, thus women were nested within the districts. Odds Ratio with 95% Confidence Intervals were used to measure the strength of association between a set of explanatory variables and utilization of skilled births attendants during delivery in multivariable multilevel logistic regression models accounting for cluster variability. *P*-value less than 5% was considered statistically significant.

#### Multilevel modelling

During the analysis, three models were run i.e. an empty model, model containing a set of covariates and the final model which was adjusted for confounders. We first estimated an “empty” model (model one), which included a random intercept and allowed us to detect the existence of any possible cluster variations on SBA use. The second model included the exposures of interest, one at a time, to determine the use of SBA for each exposure variables at a bivariate level. The last model included all variables significant at 10% (*p* ≤ 0.1) in model two, aimed to determine predictors of SBA use at a multivariable level. The final model (multivariable model) was used to predict the odds SBA use during delivery and its results were presented herewith.

## Results

### Recruitment and enrolment of participants

In total, 2844 women aged 15–49 years were recruited from all the 11 districts of Mbeya region. Out of the 2844 women, 64% (1814) reported birth in the last 5 years prior to the survey and 62% (1777) reported a live birth and 2% (37) still births in the last 5 years prior to this survey. The analysis for factors associated with skilled birth attendance use during childbirth used women who reported a live birth in previous 5 years prior to the survey i.e. 1777 women.

### Socio-demographic and reproductive health characteristics

At recruitment, the mean age (± SD) of the 1777 participants was 29 (± 7) years. Majority of the 1777 women lived in rural areas (87.5%); were married (84.9%); had primary education (74.5%); and had a monthly income of < 30 USD (80.6%). More than half of the participants (58.7%) were accessing facilities with delivery services within 30 min walking distance. In addition, most of the women had two or more children (76.2%) and attended ANC visit 4 or more times (63.4%) during the last pregnancy. Table 1 below presents these results.

**Table 1:** Socio-demographic and reproductive health characteristics of the study participants (*N* = 1777)

Variable	N	%
**Residence**		
Rural	1555	87.5
Urban	222	12.5
**Age in years (*N*** **= 1774)**		
15–24	562	31.7
25–34	783	44.1
35–49	429	24.2
**Education level**		
None	156	8.8
Primary	1324	74.5
Secondary or above	297	16.7
**Marital status**		
Married	1508	84.9
Single	125	7.0
Widow/Divorced/Separated	144	8.1
**Average level of monthly income**^**a**^		
< 30 USD	1433	80.6
≥ 30 USD	344	19.4
**Distance from home to the nearest health facility (walking)**		
Up to 30 min	1043	58.7
31 to 60 min	352	19.8
More than 60 min	382	21.5
**Number of children**		
One	424	23.9
Two	407	22.9
Three or more	946	53.3
**Gestation age at the first visit in last pregnancy**		
≤ 12 weeks	855	48.1
> 12 weeks	922	51.9
**Frequency of ANC visit at last pregnancy**		
< 4 visits	651	36.6
≥ 4 visits	1126	63.4
**Attended ANC at least once during last pregnancy**		
No	17	1.0
Yes	1760	99.0

^a^1USD =2166 Tanzania Shillings during the data collection period

### Proportion of births attended by the skilled birth attendants during delivery

Of the 1777 women who had a live birth in past 5 years prior to the survey, 81% (*n* = 1432) were assisted by a skilled birth attendant during delivery of the last child. Of the women who delivered at health facilities, nearly half delivered at the hospitals (46%), followed by dispensaries (37%). Table 2 depicts these findings.

**Table 2:** Proportion of births attended by the skilled birth attendants during delivery (*N* = 1777)

Variable	n	%
**Place of delivery of the last child**		
Health facility	1432	80.6
Home	316	17.8
Others	29	1.6
**Person who assisted at last delivery**		
Health care provider	1436	80.8
Traditional births assistant	56	3.2
Relative/mother/mother in law	208	11.7
Others/alone	77	4.3
**Level of health facility she delivered (*N*** **= 1432)**		
Dispensary	532	37.2
Health centre	240	16.8
Hospital	660	46.0

### SBA utilisation along the continuum of care

**Fig. 1:**
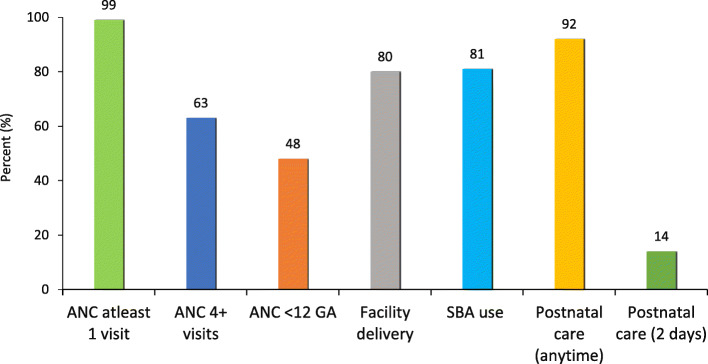
Use of maternal health services along the continuum of care (*N* = 1777)

While use of antenatal care at least once and SBA utilisation during childbirth was high, use of skilled health personnel was low at some other critical times like during early pregnancy or during immediate postpartum period as shown in Fig. 1.

### Predictors of SBA use during childbirth

Additional Files 1 and 2 presents the bivariate analyses of socio-demographic and Reproductive health factors influencing utilization of skilled birth providers during delivery. Table 3 below shows the results of multivariable multilevel regression analysis of factors influencing SBA use during delivery.

**Table 3:** Multivariable multilevel logistic regression analysis of factors influencing skilled birth attendants use during delivery

Variables	aOR^**a**^ (95% CI)	***P***- Value
**Education level**		
None	1	
Primary	1.22(0.81–1.82)	0.345
Secondary and above	2.14 (1.19–3.90)	0.012
**Distance from home to the health facility (walking)**		
Up to 30 min	1	
31 min to 60 min	0.67 (0.48–0.93)	0.012
> 60 min	0.43 (0.32–0.57)	< 0.001
**Frequency of ANC visit**		
< 4 visit	1	
≥ 4 visit	1.63 (1.26–2.10)	< 0.001
**Number of children**		
1	1	
2	0.52 (0.33–0.79)	0.003
3 and above	0.39 (0.27–0.58)	< 0.001

*aOR* Adjusted Odds Ratio

^**a**^Adjusted for age, educational level, residence, income, ANC visit, distance to health facility and number of children

Accounting for the cluster effect, SBA use was significantly higher among women having at least a secondary education (aOR = 2.14; 95% CI: 1.19–3.90; *p* = 0.012) and those who reported ANC visits for four times or more times (aOR = 1.63; 95% CI: 1.26–2.10; *p* < 0.001).

Nonetheless, SBA use was significantly lower among women having two (aOR = 0.52; 95% CI: 0.33–0.79; *p* = 0.003) or three children (aOR = 0.39; 95% CI: 0.27–0.58; *p* < 0.001) compared to those with one child; and those spending 30 to 60 min walking distance to the health facility (aOR = 0.67; 95% CI: 0.48,-0.93; *p* = 0.012) or more than 1 h walking distance to the health facility (aOR = 0.43; 95% CI: 0.32–0.57; *p* < 0.001) relative to < 30 min,.

In the null model, 9.5% of the total variance in the odds of using SBA was accounted by between cluster variability (ICC = 0.095). Nevertheless, the variability declined to7.9% in the final adjusted model making the difference of 1.6%.

### Reasons for home deliveries

**Fig. 2:**
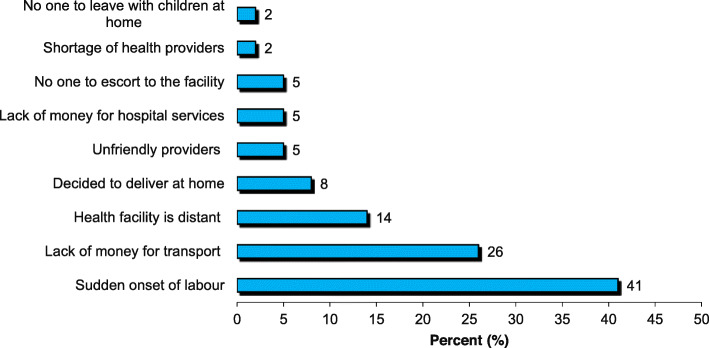
Reasons for home delivery (*N* = 316)

The reported reasons for home deliveries among 316 women who delivered at home are depicted in Fig. 2. Sudden onset of labour (41%), followed by lack of money for transport (36%) and the facility being too far (14%) were the most common reasons for home deliveries. Of importance is (8%) of the women who said they purposely decided to deliver at home, and 5% who delivered at home due to abusive and bad language of the health providers (unfriendly providers).

## Discussion

The result of this study showed high proportion of women utilising skilled birth attendant during childbirth in Mbeya Region. SBA use during childbirth was significantly higher in women with secondary education or more, and those who attended ANC visits 4 times or more. For those delivering at home, transport and associated costs were the key barrier for SBA use and institutional delivery.

The proportion of women utilising SBA during delivery in our study is higher than the national coverage (64%) [2]. In the region, SBA use during childbirth has increased from 43% in 2010, 65% in 2015 to 81% in 2016 as observed in the current study [2, 5]. The observed difference over time could be attributed by many things including efforts and interventions initiated by the Ministry of Health (MOHCDGEC) and other development partners in training and equipping facilities, raising community awareness and knowledge on the importance of being cared by a skilled provider from pregnancy throughout childbirth and postnatal period [6, 10, 24]. Mbeya region has reached the national SBA target of 80% [10, 25]. However, there is still a substantial group of women who are left behind (19%), thus efforts are required to attain and surpass the 90% goal of SBA coverage as recommended by the WHO.

Women with secondary education or higher had increased odds of SBA use during delivery compared to those with no formal education. These results corroborate the findings of a great deal of the previous studies done in Asia and Africa that showed women with secondary education or higher had significantly higher odds of utilization of skilled birth attendants during delivery compared to those with no formal education [2, 11–16]. A possible explanation for this result may be women with higher education have access to information on importance of SBA use during delivery than uneducated women [20–22]. It might also reflect more understanding of health care services or higher autonomy in decision making. However, there are other possible explanations.

Accounting for cluster effects, two strong markers of inequity in SBA use during childbirth (i.e. urban residence and higher monthly income) were not associated with SBA use in this study. These findings were unexpected and contradicts with studies conducted in Asia, sub-Saharan Africa and particularly Tanzania [2, 5, 15–22]. Nevertheless, there was still 12% difference in SBA use during childbirth between women living in urban (91%) and rural (79%) areas in this setting. These findings highlight the need for continued input of resources, efforts of raising community awareness in SBA use and equipping health facilities (mainly dispensaries and health centres) to offer quality care in rural areas of the region [26].

Distance to the health facility was another significant predictor of SBA use in this study. Most women who were staying within 30 min walking distance to access health facility (86%) utilised SBA during childbirth than those spending longer duration (69%). Other studies reported that living within 60 min walking distance to health facility [2, 16] and living within 2 to 5 km to the health facility with delivery services increased the odds of SBA use during childbirth [14, 21, 22, 27]. There are two possible explanations for this; the transport fare to reach health facilities that are far is a limiting factor [14, 21, 22]; and/or the physical terrain restricts them from accessing health facilities [27]. However, the autonomy of seeking care and dependence on partners may confound this association.

Transport was a key barrier reported by many women who delivered at home. There were two scenarios regarding transport. First women reported that when labour pains start at night, it is difficult to organize or get transport (even getting motorcycles which are commonly available even in the rural settings). Additionally, women reported that, even if you get transport at night, charges are extremely high, sometimes up to 3–4 times higher than what they will be charged during the daytime. The second scenario was that, nearly 4 out of ten women who delivered at home reported lack of money to pay for transport as a key barrier. Some just gave up because the facility with delivery services is far. The results are similar to previous studies done in Tanzania, Africa and Asia [2, 10, 19, 21, 22]. This calls for the district managers to revive or strengthen the village health teams and work together in coming with local-based solutions in transport for women in labour especially those who live far away from health facilities and in rural areas.

Of importance in Mbeya region was a group of women (8%) among those who delivered at home who said they purposely decided and planned to deliver at home. It maybe that these women perceived they have low risk of having complicated childbirth because they had pregnancies that did not have any problems [22]. However, emergencies may still arise since every pregnancy is at risk and 15% of pregnancies may end up with life threatening complications. The need to educate women on danger signs and importance of accessing the closest health facility that can handle complications needs to be emphasized during antenatal care. Another driving force on deciding to deliver at home might be the previous bad experience with providers at the facilities as reported by others. Studies have shown that women are afraid of the abusive language or harsh treatment of providers remembering the last time they delivered at facilities and they would rather deliver with relative support rather than repeating the experience [10, 13, 21, 22]. The need to have respectful care and maintaining women’s dignity is a strong component of quality of care that needs to be improved in every childbirth in Tanzania.

In this study, attending ANC 4 times or more was associated with increased SBA use at childbirth. Similar finding was reported by studies conducted in Afghanistan, India, Nigeria, Ethiopia, Kenya, Uganda and Tanzania (Mtwara region) [2, 10, 13, 15–19, 21, 22]. Evidence from previous studies indicated that, attending recommended number of ANC visits provide women with a chance to receive more counselling and key interventions [14, 21, 27]. Furthermore, high frequency of contacts with health facility and healthcare providers improve women trust to providers which in turn increases women’s likelihood to use SBA during childbirth [15, 28]. Currently, it is recommended by the WHO that, women should have a minimum of 8 visits with skilled health personnel during pregnancy for positive outcomes and experience; five of those contacts should be in the third trimester [29]. Therefore, the need for health providers to encourage women to start ANC early (≤ 12 weeks) and attend recommended times cannot be overemphasized.

As depicted by the literature that SBA use plays important roles in reducing preventable maternal deaths. In Tanzania and Mbeya region in particular, while SBA use during childbirth has increased over time this is opposite to what is reported for maternal deaths [2, 5]. Can this be explained by missing opportunity to offer comprehensive care by the health system at all critical points in the continuum of care as shown in this study i.e. low attendance for postnatal care within 2 days after delivery? [4, 30]. Or it may be explained by the fact that, while many SSA governments like Tanzania have increased the number of training institutions and enrolment of health professionals in order to increase the number of trained health providers who are reported as SBAs, but did not improve their actual skills and competencies for saving maternal and newborn lives as per FIGO, ICM or ICN standards criteria of competence? [9]. Provision of quality care immediately after delivery is vital to avert nearly half of maternal and newborn deaths [1, 4, 9, 30]. Measurement of SBA coverage during deliveries is challenging [7, 9]. In 2018, WHO, UNFPA, UNICEF, ICM, ICN, FIGO and IPA have refined the 2004 definition of “skilled health personnel” hoping it will help in truly capturing providers who are skilled health personnel [8, 9].

The key strengths of this study include its large and representative sample of women of reproductive age in the region. The information obtained can be generalized to the whole region. Despite the strengths, generalisability of these results is subject to certain limitations. The study utilized women with a live birth in 5 years prior to the study to assess prevalence and factors associated with SBA use. It has missing those who had a negative pregnancy outcome or ended with maternal death. It might be that those with negative outcomes were less likely to use SBA and thus overestimating the SBA use. Future studies will need to use robust designs that can show the association between SBA use in continuum of care and pregnancy outcomes including maternal and newborn deaths. SBA use during childbirth was based on women self-reports. However, it is difficult for some women to differentiate various cadres of health providers they meet at the facilities. This can happen when a woman was assisted by a person other than SBA (according to the WHO definition) e.g. nurse attendants, leading to higher estimate of SBA use. Nevertheless, the results of this study are not likely to be affected by this since multiple questions were used to probe the woman in order to identify appropriate cadre of the provider.

## Conclusion

The prevalence of births attended by SBA during delivery was high (81%) in this setting. Considering cluster variability, having at least secondary education and attending four or more ANC visits increased SBA use during childbirth whereas long distance to the health facility and having more than one child decreased SBA use. Based on these findings, we recommend the following: -

Healthcare providers need to encourage women to attend the recommended ANC visits to improve maternal and newborn outcomes. Hence, efforts beyond the health system are needed to reach uneducated women and older pregnant mothers aged more than 35 years
The need to educate women on their expected date of birth is important and innovative technologies of measuring EDD are required in this setting. This will improve preparedness and avoid the reports of home deliveries due to sudden onset of labour.
Efforts to improve the quality of ANC and SBA is also necessary for improvement in maternal or newborn health outcomes in the Region.
Qualitative study to explore the barriers of SBA use among the 19% who are not using skilled assistance during childbirth is needed.

## Supplementary information

**Additional file 1 **The Socio-demographic factors influencing the utilization of skilled births attendants during delivery (*N* = 1777).

**Additional file 2 **Reproductive health factors influencing utilization of skilled births attendants during delivery (*N* = 1777).

**Additional file 3.** Questionnaire.

## Data Availability

Due to ongoing analyses, the supporting data are available from the corresponding author on a reasonable request.

## Abbreviations

ANC: Antenatal Care
aOR: Adjusted Odds Ratio
CI: Confidence Interval
ICC: Intra-cluster Correlation Coefficient
NBS: National Bureau of Statistics
MDG: Millennium development Goal
MMR: Maternal Mortality Ratio
OR: Odds Ratio
SBA: Skilled Birth Attendant
SDG: Sustainable Development Goal
WRA: Women of Reproductive Age
WHO: World Health Organization
