# Supplementary material for: Factors influencing utilization of skilled birth attendant during childbirth in the Southern highlands, Tanzania: a multilevel analysis
Source: BMC Pregnancy Childbirth. 2020 Jul 25;20:420. doi: 10.1186/s12884-020-03110-8 (PMC7382099; doi:10.1186/s12884-020-03110-8)
Supplement: Supplementary file 1 — Additional file 1 The Socio-demographic factors influencing the utilization of skilled births attendants during delivery (N = 1777). [file 12884_2020_3110_MOESM1_ESM.docx]

**Additional File 1: The Socio-demographic factors influencing the utilization of skilled births attendants during delivery (N=1777)**

|  |  | | **SBA use at last delivery** | **Crude** |  | |
| --- | --- | --- | --- | --- | --- | --- |
| **Variables** | **N** | | **n (%)** | **OR (95% CI)** | **P-value** | |
| **Residence** | |  |  |  | |  |
| Rural | | 1555 | 1235 (79.4) | 1 | |  |
| Urban | | 222 | 201 (90.5) | 1.66 (0.97-2.83) | | 0.064 |
| **Age groups (years)** | |  |  |  | |  |
| 15-24 | | 562 | 483 (85.9) | 1 | |  |
| 25-34 | | 783 | 628 (80.2) | 0.68 (0.50-0.91) | | 0.010 |
| 35-49 | | 429 | 323 (75.3) | 0.50 (0.35-0.68) | | 0.012 |
| **Education level** | |  |  |  | |  |
| None | | 156 | 115 (73.7) | 1 | |  |
| Primary | | 1324 | 1049 (79.2) | 1.29 (0.87-1.92) | | 0.201 |
| Secondary and above | | 297 | 272 (91.6) | 3.27 (1.87-5.73) | | <0.001 |
| **Marital status** | |  |  |  | |  |
| Married | | 1508 | 1218 (80.8) | 1 | |  |
| Single | | 125 | 107 (85.6) | 1.34 (0.67-1.93) | | 0.642 |
| Divorced/widow/separated | | 144 | 111 (77.1) | 0.74 (0.49-1.13) | | 0.178 |
| **Participant Income level per month** ⱡ | |  |  |  | |  |
| <30USD | | 1433 | 1137 (79.3) | 1 | |  |
| ≥30USD | | 344 | 229 (86.9) | 1.65 (1.16-2.33) | | 0.005 |
| **Distance from home to the health facility (walking)** | |  |  |  | |  |
| Up to 30 minutes | | 1043 | 893 (85.6) | 1 | |  |
| 31 minutes to 60 minutes | | 352 | 280 (79.5) | 0.67 (0.49-0.92) | | 0.015 |
| >60 minutes | | 382 | 263 (68.8) | 0.41 (0.31-0.55) | | <0.001 |

*ⱡ 1USD = 2166 Tanzania Shillings during data collection*
