# Supplementary material for: Factors influencing utilization of skilled birth attendant during childbirth in the Southern highlands, Tanzania: a multilevel analysis
Source: BMC Pregnancy Childbirth. 2020 Jul 25;20:420. doi: 10.1186/s12884-020-03110-8 (PMC7382099; doi:10.1186/s12884-020-03110-8)
Supplement: Supplementary file 2 — Additional file 2 Reproductive health factors influencing utilization of skilled births attendants during delivery (N = 1777). [file 12884_2020_3110_MOESM2_ESM.docx]

Additional File 2: Reproductive health factors influencing utilization of skilled births attendants during delivery (N=1777)

|  | |  | **SBA use at last delivery** | | **Crude** |  |
| --- | --- | --- | --- | --- | --- | --- |
| **Variables** | **N** | | | **n (%)** | **OR (95% CI)** | **P-value** |
| **Participant gestation age at the first ANC visit** | | | | |  |  |
| ≤12 weeks | 855 | | | 705 (82.5) | 1 |  |
| >12 weeks | 922 | | | 731 (79.3) | 0.81 (0.64-1.03) | 0.091 |
| **Frequency of ANC visit** |  | | |  |  |  |
| <4 visit | 651 | | | 494 (75.9) | 1 |  |
| ≥4 visit | 1126 | | | 942 (83.7) | 1.65 (1.29-2.11) | <0.001 |
| **Attended for ANC at least once during pregnancy** |  | | |  |  |  |
| No | 17 | | | 8 (47.1) | 1 |  |
| Yes | 1760 | | | 1428 (81.1) | 4.79 (1.75-13.14) | 0.002 |
| **Number of children** |  | | |  |  |  |
| 1 | 424 | | | 384 (90.6) | 1 |  |
| 2 | 407 | | | 336 (82.6) | 0.49 (0.32-0.75) | <0.001 |
| 3 and above | 946 | | | 716 (75.6) | 0.33 (0.23-0.48) | <0.001 |
| **Knowledge of danger signs during pregnancy** |  | | |  |  |  |
| Poor knowledge (<3) | 1624 | | | 1302 (80.2) | 1 |  |
| Good knowledge (≥3) | 153 | | | 134 (87.6) | 1.57 (0.95 -2.61) | 0.080 |
| **Knowledge of danger signs during delivery** |  | | |  |  |  |
| Poor knowledge (<3) | 1709 | | | 1375(80.5) | 1 |  |
| Good knowledge (≥3) | 68 | | | 61 (89.7) | 1.84 (0.82-4.11) | 0.139 |
| **Knowledge of danger signs after delivery** |  | | |  |  |  |
| Poor knowledge (<3) | 1696 | | | 1364 (80.4) | 1 |  |
| Good knowledge (≥3) | 81 | | | 72 (88.9) | 1.64 (0.80-3.36) | 0.178 |
| **Had Birth preparedness and complication readiness plan** |  | | |  |  |  |
| Not well prepared (<3) | 1543 | | | 1235 (80.0) | 1 |  |
| Well prepared (≥3) | 234 | | | 201 (85.9) | 1.41 (1.95-2.11) | 0.091 |
