# Supplementary material for: Factors influencing utilization of skilled birth attendant during childbirth in the Southern highlands, Tanzania: a multilevel analysis
Source: BMC Pregnancy Childbirth. 2020 Jul 25;20:420. doi: 10.1186/s12884-020-03110-8 (PMC7382099; doi:10.1186/s12884-020-03110-8)
Supplement: Supplementary file 3 — Additional file 3. Questionnaire. [file 12884_2020_3110_MOESM3_ESM.pdf]

QUESTIONNAIRE FOR WOMEN OF REPRODUCTIVE AGE

Dododoso la akina mama walio katika umri wa kuzaa

KNOWLEDGE, AWARENESS AND UTILIZATION OF SKILLED BIRTH ATTENDANTS FOR RMNCH SERVICES AMONG WOMEN (15-49 YEARS) IN MBEYA REGION, TANZANIA.

**ID of participant:** *Namba* ………………………….. **Nearest health facility:** *Jina la kituo cha afya kilicho jirani* ………………................................ **Level of nearest HF:** *Ngazi ya kituo* ……………………………..	**Name of district:** *Wilaya* ………………………………. **Name of ward:** *Kata* ………………………………. **Name of village/street:***kijiji/mtaa* …………………………..……. **Urban or rural:** *mjini/kijiji* ……………………………….	**Date of interview:** *Tarehe ya mahojiano* ……………………………………………….. **Name of interviewer:** *Jina la anayehoji* ………………………………………………… **Tel of interview:** *Namba ya simu ya anayehojiwa* ………………………………………………… **Date of Last Live Delivery:** *Tarehe ya kujifungua mtoto hai mara ya mwisho* **(dd/mm/yyyy)**……………

I. Socio – Demographic Characteristics

1. Date of birth of woman in years *Tarehe ya kuzaliwa (DD MM YYYY)*……………….……….

2. Age of the participant in years *Umri jaza miaka iliyokamilika*….………………………………

3. Marital status *Hali yako ya ndoa kwa sasa ikoje?*

1. Married *Nimeolewa*
2. Cohabiting *Tunaishi bila ndoa*
3. Single *Sijawahi kuolewa*
4. Divorced/Separated *Talaka/Tumetengana*
5. Widow *Mjane*

4. (a) If Married/Cohabiting for how many years are you living together *Kama umeolewa au kuishi bila ndoa, je mmeishi pamaoja na mwenzi kwa muda gani?*………………….…………….

(b) Separated/Divorced/Widow for how many years *Kama ni mmetengana, mtalaka au mjane umekaa hivyo kwa muda gani?*…………………………….……………………………………….….

5. Education level *Kiwango cha elimu cha mshiriki*

1. Never been in school *Hakusoma*
2. Primary Education *Elimu ya msingi*
3. Secondary Education (''O'' or ''A'' level) *Elimu ya sekondari*
4. Tertiary Education *Elimu ya juu*

6. Are you employed and you receive regular month salary? *Je umeajiriwa na unapokea mshahara kila mwisho wa mwezi?*

0. No *Hapana*
1. Yes *Ndiyo*

7. Occupation *Kazi*

1. Civil Servant *Mtumishi wa serikali*
2. House wife *Mama wa nyumbani*
3. Peasant *Mkulima mdogo*
4. Small business *Biashara ndogondogo*
5. Other specify……………………………………………….…………

Damian et al, 2020

8. What is the age of your partner? *Umri wa mwenzi*

Age in years *Umri kwa miaka*……………………….……………

0. Don't know *Sijui umri wake*

9. Partners' education level *Kiwango cha elimu cha mwenzi*

0. Don't know *Sijui kiwango chake cha elimu*
1. Never been in school *Hajawahi kusoma*
2. Primary Education *Elimu ya msingi*
3. Secondary Education (''O'' or ''A'' level) *Elimu ya sekondari*
4. Tertiary Education – degree/advanced diploma *Elimu ya juu*

10. Partners' employment status *Kazi ya mwenzi*

1. Employed with monthly salary *Ameajiriwa na anapokea mshahara kila mwisho wa mwezi*
2. Self employed, type of job *Amejiariri, taja kazi*..........……………..
3. Trader *Biashara ndogo ndogo*
4. Business man *Biashara kubwa*
5. Laborer *Vibarua*
6. Others please specify …………………………….....……………….

11. Living arrangement *Mnavyoishi na mwenza*

1. Living with partner *Tunaishi pamoja*
2. Visits *Ananitembelea*
3. Lives apart > 3 months *Tunaishi mbali mbali zaidi ya miezi 3*

12. Regarding accommodation, are you living in your own house or rented? *Je mnaishi/unaishi kwenye nyumba ya kupanga au nyumba yenu wenyewe?*

1. Live in our own house *Tunaishi kwenye nyumba yetu wenyewe* – 13a
2. Rented house *Tumepangisha* ----- go to 13 b

13a. If you own a house, type *Kama ni nyumba yeni, je ni;*

1. Brick house *Ni nyumba ya tofali*
2. Mud house *Ya udongo/ tepe*

13 b. If you are renting; are you renting a whole house, some rooms or a room? *Kama mnakodi;*

1. Rent a whole house *Tumekodi nyumba nzima*
2. Rent rooms *Tumekodisha vyumba taja idadi…………*
3. Have rented one room *Tu/nimekodi chumba kimoja*

14. Approximate income of the woman per month? *Kwa kukadiria kipato chako kwa mwezi ni kiasi gani?* Tanzanian shs ……………………………….*(If doesn't have record 0)*

15. What is approximate family income per month (yours partner plus your income)? *Kwa kukadiria je kipato cha familia ni kiasi gani ukiweka chako na cha mwenzi?*

Tanzanian shs……………………………….

Damian et al, 2020

16a. What is the nearest health facility with antenatal and child health services? *Kliniki ya karibu inayotoa huduma za mama na mtoto?* Name……………………………………………

Is it a

1. Dispensary *Dispensari/zahanati*
2. Health centre *Kituo cha afya*
3. Hospital? *Hospitali*

16b. Can you mention all the reproductive, maternal, newborn and child health services which you are aware a woman can get at nearest health facility mentioned in Q 16a? *Je ni huduma zipi za uzazi, mama na watoto zilizo katika kliniki iliyo karibu na wewe?*..

……

……

16C. Of the mentioned reproductive, maternal, newborn and child health services in Q 16b, which ones have you used in the past 5 years? *Je katika huduma ulizotaja swali 16b, ni huduma zipi za uzazi, mama na watoto zilizo katika kliniki iliyo karibu na wewe ulizotumia au kupata katika kipindi cha miaka 5 iliyopita?*……

……

17. What is the nearest health facility with delivery services? *Kliniki ya karibu inayotoa huduma za kujifungua* Name……………………………………

Is it a

1. Dispensary *Dispensari/zahanati*
2. Health centre *Kituo cha afya*
3. Hospital? *Hospitali*

b) What is the distance from home to nearest health facility with delivery services when walking? *Je ukitembea kwa miguu inakuchukua muda gani toka kwako hadi ufike kituo cha karibu chenye huduma za kuzalisha?*

1. Up to 30 minutes *hadi dakika 30*
2. 31 minutes to 60 minutes *dakika 31 hadi 60*
3. 61 – 120 minutes (> 1 hour to 2 hours) *dakika 61 hadi 120*
4. 121 minutes – 180 minutes (> 2 hours to 3 hours) *dakika 121 hadi 180*
5. > 181 minutes (More than 3 hours) *zaidi ya dakika 180*

c) What is the distance from home to the nearest health facility with delivery services when using a car or motorcycle? *Ukitumia pikipiki au gari, je kuna umbali gani toka kwako hadi ufike kituo cha karibu chenye huduma za kuzalisha?*

1. Up to 30 minutes *hadi dakika 30*
2. 31 minutes to 60 minutes *dakika 31 hadi 60*
3. 61 – 120 minutes (> 1 hour to 2 hours) *dakika 61 hadi 120*
4. 121 minutes – 180 minutes (> 2 hours to 3 hours) *dakika 121 hadi 180*
5. > 181 minutes (More than 3 hours) *zaidi ya dakika 180*

Damian et al, 2020

d). What is the fare you use to pay from home to the nearest health facility with delivery services when using a car or motorcycle? *Je unalipa kiasi gani ukitumia pikipiki au gari, ili ufike kwenye kituo cha afya cha karibu chenye huduma za kuzalisha?*

1. Fare by using motor cycle in TZS *Nauli kwa pikipiki*…………………………………………………
2. Fare by using public transport (hiace) in TZS *Nauli kwa kibasi*……..…………………………..
3. Fare by using hired car in TZS *Nauli kwa taxi au gari la kukodi*……………………………….

e). What is the common mode of transport do most women use when they go for delivery at your area? *Kwa kawaida usafiri gani unatumiwa na wamawake walio wengi wanapokwenda kujifungua?* ……….

II. Reproductive & Maternal health history

18. a) When was your last delivery (DD, MM, YYYY) *Mara ya mwisho ulijifungua lini? ……………….*

b) Age of your last born *Umri wa mtoto wa mwisho kwa* ***miezi*** ……………………………………………

c) Number of pregnancies *Umeshawahi kuwa mjamzito mara ngapi?*……………………………………

d) How many children are alive *Je watoto wangapi walio hai?*....……………………....................

e) How many pregnancies ended with miscarriage/abortion? *Mimba kuharibika.*………………..

f) How many pregnancies ended with stillbirth? *Mtoto alizaliwa akiwa mfu?*…...…………………

g) List all the deliveries the woman had ever had start from the first to the last born *Jaza jedwali hapo chini ya watoto wote ambao mama ameshawahi kuzaa ukianzia wa kwanza hadi wa mwisho*

**S/N**	**Birth order**	**DOB (dd/mm/yyyy)** *Tarehe ya kuzaliwa*	**Sex** *Jinsia*	**Place of delivery (home vs. health facility)** *Sehemu alipojifungulia nyumbani au kituo cha afya?*	**Who assisted in delivery** *Nani alimsaidia au kumzalisha?*
	1st born				
	2nd born				
	3rd born				
	4th born				
	5th born				
	6th born				
	7th born				

Summarize: Total number of **births** in the past 5 years *Jumla ya watoto aliojifungua kwa miaka mitano iliyopita* ………………………………………

Total number of **live births** in the past 5 years *Jumla ya watoto walio hai aliojifungua kwa miaka mitano iliyopita* ……………………………………

Information on last pregnancy *Taarifa kuhusu ujauzito wa mwisho*

19. Did you attend/go for antenatal care/ clinic during your **last pregnancy?** *Ulihudhuria kliniki kwenye ujauzito wako wa mwhisho?*

Damian et al. 2020

0. No
1. Yes

19.2: What was the gestation age on the first visit? *Mimba ilikuwa na umri gani* ***kwa miezi*** *ulipohudhuria kliniki kwa mara ya kwanza?* ………………………………………………………………………….

19.3: How many times did you attend for antenatal care? *Ulihudhuria kliniki mara ngapi hadi ulipojifungua?* ……………………………………………………………..…………………….

19.4: Who was attending you most of the times when you attended for ANC? *Nani alikuwa anakupa huduma mara kwa mara katika ujauzito wako wa mwisho?*

1. Nurse/ midwife
2. Doctor
3. Other please mention………………………..……………………………………..

19.5: Any problem detected during pregnancy? *Je kuna shida yeyote iligundulika wakati wa ujauzito?*……………………………………………………………………………………….………………………………..

19.6: During antenatal care visits of **your last pregnancy** you were told about things to look for/ or to know, that may suggest a woman has problems and she needs to seek care? *Wakati wa ujauzito wako wa mwisho, je uliambiwa mambo ambayo mjamzito anayopaswa kuzingatia au kuangalia/ kuchunguza ili kwa kuziona dalili hizo ajue ana matatizo ya ujauzito ili kupata huduma?* 0. No
1. Yes

19.7: Can you tell me which key danger signs will suggest problems <u>during pregnancy</u> until a woman has to seek care? (*do not prompt circle all mentioned) Unaweza kuniambia dalili za hatari katika <u>ujauzito</u> zinazohitaji kupatiwa huduma?* ***(Usimtajie, Zungushia zote atakazozitajia)***

Vaginal bleeding *kutoka damu kwenye uke* 0. No 1. Yes 2. Don't know
Reduced/cessation of fetal movements *kupungua au mtoto kutocheza tumboni* 0. No 1. Yes 2. Don't know
Swollen hands/legs *miguu na mikono kuvimba* 0. No 1. Yes 2. Don't know
Severe headache *kichwa kuuma sana* 0. No 1. Yes 2. Don't know
Blurred vision *kutoona vizuri* 0. No 1. Yes 2. Don't know
Fits/ convulsions *degedege* 0. No 1. Yes 2. Don't know
Others *nyinginezo taja* …………………………………………………..

………
……….

19.8: Can you tell me which key danger signs or problems to look for <u>during labour and childbirth</u> that will necessitate the woman or her family to seek care? (*do not prompt circle all mentioned) Je, unaweza kunaimbia dalili hatarishi wakati wa <u>kujifungua</u> zinazo walazimu wanafamilia kutafuta huduma ya afya?**(* ***Usimtajie, Zungushia zote kwa kadri atakavyotaja)***

Severe vaginal bleeding *kutoka damu nyingi sana ukeni* 0. No 1. Yes 2. DK

Damian et al., 2020

Prolonged labour (> 12 hours) *kukaa kwenye hali ya kujifungua zaidi ya masaa 12*
0. No 1. Yes 2. DK
Fits/ convulsions *degedege* 0. No 1. Yes 2. DK
Retained placenta *kubakia kwa kondo la nyuma* 0. No 1. Yes 2. DK
Others
nyinginezo…………………………………………………………………………………………………

19.9: Can you tell me which key danger signs or problems to look for after delivery that will necessitate the woman or her family to seek care? (*do not prompt circle all mentioned) Je, unaweza kunaimbia dalili hatarishi baada ya kujifungua zinazo walazimu wanafamilia kutafuta huduma ya afya?**(Usimtajie, Zungushia zote kwa kadri atakavyotaja)***

Severe vaginal bleeding *kutoka damu nyingi sana ukeni* 0. No 1. Yes 2. DK
Foul smelling vaginal discharge *kutoka uchafu ukeni au harufu mbaya*
0. No 1. Yes 2. DK
High fever *homa kali* 0. No 1. Yes 2. DK
Fits/ convulsions *degedege* 0. No 1. Yes 2. DK
Others
nyinginezo…………………………………………………………………………………………………

19.10: Were you counseled on the following issues during your **last pregnancy**? *Je, katika ujauzito wako wa mwisho ulipewa ushauri na wataalamu wa afya kuhusu mambo yafuatayo?*

HIV counseling and Testing<br>*Kuhusu kupima VVU wakati wa ujauzito*	0. No	1. Yes	2. Don't know
Danger signs of pregnancy<br>*Dalili hatari za ujauzito*	0. No	1. Yes	2. Don't know
Danger sign during C/birth<br>*Dalili hatari wakati wa kujifungua*	0. No	1. Yes	2. Don't know
Danger signs of the neonate<br>*Dalili hatari kwa mtoto mchanga*	0. No	1. Yes	2. Don't know
On birth preparedness<br>*Utayarishaji wa kujifungua*	0. No	1. Yes	2. Don't know
Obstetric complications<br>*Matatizo ya uzazi*	0. No	1. Yes	2. Don't know

19.11: Were the following measured or performed during your **last pregnancy**? *Je vipimo vifuatavyo vilifanyika wakatiwa ujauzito wako wa mwisho?*

Blood pressure measured<br>*Kupimwa shinikizo la damu*	0. No	1. Yes	2. Don't know
Weight taken<br>*Kupimwa uzito*	0. No	1. Yes	2. Don't know
Blood checked for			
HIV<br>*Kupima virusi vya UKIMWI*	0. No	1. Yes	2. Don't know
Syphilis<br>*Kupimwa kaswende*	0. No	1. Yes	2. Don't know
Hemoglobin<br>*Kupimwa wingi wa damu*	0. No	1. Yes	2. Don't know
Urine taken<br>*Kupimwa mkojo*	0. No	1. Yes	2. Don't know

Damian et al, 2020

Given SP (IPT) to prevent malaria 0. No 1. Yes 2. Don't know

Alipewa Sp (IPT) kuzuia malaria

If given IPT how many times?.Kama alipewa ni mara ngapi?...

19.12: Did you have a birth plan in your last pregnancy? *Je ulifanya maandalizi au ulijitayarisha kwa kujifingua katika ujauzito wa mwisho?*

0. No 1. Yes 2. Don't know what is birth plan

19.13: Which level of care did you get your antenatal care for the last pregnancy? *Je ni kituo kipi ambacho ulipata huduma za afya katika ujauzito wa mwisho?*

1. Dispensary *Dispensari/zahanati* (Name *Jina*…………………………………)
2. Health Center *Kituo cha afya* (Name *Jina*…………………..…………………)
3. Hospital *Hospitali* (Name *Jina*…………………………………………………..…)

19.14: Were you satisfied with care you received during your last pregnancy? *Je, uliridhishwa na huduma uliyopewa katika kituo cha afya kwenye ujauzito wako wa mwisho?*

0. No *Hapana (sababu za kutoridishwa?...)*
1. Yes *Ndiyo*

19.15: In general when/ at what gestation age do the women start to attend for antenatal care in this area? *Kwa kawaida katika eneo hili, wanawake wanaanza kuhudhuria kliniki mimba ikiwa na umri gani?...*

19.16: In your understanding when or at what gestation age should a pregnant woman start to attend for antenatal care? *Kwa uelewa wako je mama mjamzito anatakiwa aanze kliniki mimba ikiwa na umri gani/ miezi mingapi?...*

Place of delivery for last pregnancy *Alipojifungulia ujauzito wa mwisho*

20: Where did you give birth of your last baby? (*last born) Je mtoto wa mwisho ulijifungulia wapi?*

1. Health facility *Kituo cha afya*
2. Home *Nyumbani*
3. Other *Kwingine*………………………………………………………………………………

20.2: Who assisted with delivery of your last child? *Nani alikuzalisha?*

1. Health Care provider *Mtaalamu wa afya*
2. TBA *Mkunga wa jadi*
3. Relative/Mother/Mother in law *Ndugu/mama mzazi/mama mkwe*
4. Others mention *Wengine taja*……………………………………………………………….

20.3: Did you get any problems/complications during delivery of your last born child? *Je, ulikutwa na matatizo yeyote wakati wa kujifungua katika ujauzito wa mtoto wako wa mwisho?*

0. No
1. Yes (*please specify*………..…………………………………………………)

Damian et al, 2020

20.3b: Did the newborn get any problems/complications immediately after delivery? *Je, Mtoto alipata shida yeyote ya kiafya mara tu baada ya kuzaliwa?*

0. No
1. Yes (*please specify……..………………………………………………)*

20.4: After delivery of your last child, how long did you stay at the hospital before discharge? Je b*aada ya kujifungua mtoto wako wa mwisho, ulikaa hospitali au kituo cha afya kwa muda gani kabla ya kuruhusiwa kwenda nyumbani?*

No of hours or days......………………………………………………………….

0. Not applicable delivered at home

20.5: Before you were discharged, were you counseled on danger signs which a woman can get after delivery that will require coming to the health facility? *Kabla ya kuruhusiwa kwenda nyumbani, je ulipewa ushauri juu ya dalili za hatari ambazo mwanamke anaweza kuzipata baada ya kujifungua ambazo akiziona ni muhimu aende kupata huduma katika kituo cha kutolea huduma?* 0. No

1. Yes
2. Not applicable because delivered at home

20.6: Before you were discharged, were you counseled on danger signs for a neonate/newborn? *Kabla ya kuruhusiwa kwenda nyumbani, je ulipewa ushauri juu ya dalili za hatari za mtoto aliyezaliwa?*

0. No
1. Yes
2. Not applicable because delivered at home

20.7: Which level of health facility did you deliver your last baby? *Jinan a ngazi ya kituo alichojifungulia mtoto wa mwisho*

1. Dispensary *Dispensari/zahanati* (Name *Jina*…………………………………)
2. Health Center *Kituo cha afya* (Name *Jina*…………………..…………………)
3. Hospital *Hospitali* (Name *Jina*…………………………………………………..…)
4. Not applicable because delivered at home *alijifungulia nyumbani*

20.8: Were you satisfied with care you received during delivery at the health facility? *Je uliridhishwa na huduma ulizopewa katika kituo ulichojifungulia mtoto wako wa mwisho?*

1. Yes
0. No (*why not?...)*
2. Not applicable delivered at home *Alijifungulia nyumbani haihusiki*

20.9: Why <u>did you deliver your last baby</u> at home? *Ni kwanini uliamua kujifungulia nyumbani?*

……
……
………………………………………………

21. Did you go/attend for post-natal check up at the health facility after delivery of **your last born** baby? *Je, ulihudhuria kituo cha afya ili kupata huduma baada ya kujifungua mtoto wako wa mwisho?* 0. No

1. Yes

Damian et al, 2020

21.2: How long after delivery did you go for 1st check up? *Ulichukua muda gani baada ya kujifungua kwenda kupata huduma kliniki kwa mara ya kwanza? ....*days

21.3: How frequent did you attend for post-natal care from delivery to 40 days post partum? *Je ni mara ngapi umehudhuria huduma za kliniki ndani ya siku 40 baada ya kujifungua?*

1. Once/*Mara moja*
2. Twice/ *Mara mbili*
3. Three or more/ *Zaidi ya mara tatu*

21.4: Where did you attend for check up? *Ulikwenda wapi kwa ajili ya ufuatiliaji wa afya yako?*

1. Dispensary/*Zahanati (*Name/ *Jina…………………………………………………………)*
2. Health Center/*Kituo cha Afya (*Name/*Jina ……………………………………………)*
3. Hospital / *Hospital (*Name/*Jina ………………………………………………………)*

21.5: Which care/services; were you given yourself ? *Wewe binafsi ulipewa huduma gani?* ………

Services given for the baby? *Mtoto alipewa huduma gani?*.....................................

………

21.6: In general, what were you counseled about during postnatal care visits? *Je unapokuja kliniki baada ya kujifungua umeshapewa ushauri kuhusu mada zipi?*..

……

III. Now I will ask you some questions about birth preparedness *Sasa nitakuuliza maswali machache juu ya maandalizi ya kujifungua*

22. During your last pregnancy, were you counseled or heard about birth preparedness? *Je, wakati wa ujauzito wako wa mwisho, je ulipata ushauri au kusikia juu ya maandalizi ya kujifungua?*

0. No 1. Yes

22.2: What do you understand by IBP (Individual birth plan) *Unafahamu au unaelewa nini juu ya mpango binafsi wa kujifungua*

………

………

22.3: What were important things you were told to have in your individual birth plan (IBP)? (*do not prompt circle all mentioned) Vitu gani vya muhimu ulivyoambiwa unapaswa kujitayarisha katika mpango binafsi wa kujifungua?*

a) Choose health facility of delivery 0. No 1. Yes 2. Don't know

Damian et al, 2020

Kuchagua kituo cha afya cha kujifungua
b) Have a provider who will help with delivery 0. No 1. Yes 2. Don't know
Kuchagua mtaalamu atakayesaidia wakati wa kujifungua
c) Prepare money for transport when labor start 0. No 1. Yes 2. Don't know
Kuandaa pesa kwa ajili ya usafiri uchungu ukianza
d) Prepare transport in case of emergency 0. No 1. Yes 2. Don't know
Kuandaa usafiri iwapo kutakuwa na tatizo/ dalili za hatari
e) Prepare money in case operation needed 0. No 1. Yes 2. Don't know
Kutayarisha pesa iwapo upasuaji utahitajika
f) Prepare potential blood donors if needed 0. No 1. Yes 2. Don't know
Kutayarisha watu watakaotoa damu
g) Others *Matayarisho mengine, taja* ……………………………………………………………………
……

23. Please can you tell us which things are needed that may enhance or make women want to deliver at the health facilities in this community/area?. *Tafadhali unaweza kueleza mambo yanayotakiwa kufanyika au kuboreshwa katika jamii hii/ eneo hili ili kuwawezesha akina mama kujifungulia katika vituo vya afya?*
……
……
……

24. Kindly describe the possible barriers to health facility delivery in your community/this area.
Je kuna vikwazo gani vinavyofanya iwe vigumu kwa wamama wajawazito kwenda kujifungua kwenye vituo vya afya katika eneo hili?
……
……
……
……………………………………………………………………………

<u>IV. Future use of health facilities *Matumizi ya vituo vya afya kwa mimba zijazo*</u>

25. Are you planning to have more babies? *Je una mpango wa kupata watoto wengine?*
0. No 1. Yes

26 a. Will you attend for ANC at the facility in case you are blessed with another pregnancy? *Je utahudhuria kliniki iwapo utajaliwa ujauzito mwingine?*
0. No 1. Yes

26 b. Will you attend for ANC in the same facility like last pregnancy? *Je utahudhuria kliniki ile ile uliyopata huduma katika ujauzito wako wa mwisho?*
0. No 1. Yes

Give reason for your answer *Fafanua*…………………………………………………………………………………

Damian et al, 2020

27. Where would you like to **deliver** your baby in the next pregnancy? *Ungependa kujifungulia wapi iwapo utajaliwa ujauzito mwingine?*……………………………………………………………………………………

27b. Will you like to use the health facility for delivery of your next baby? *Je ungependa kujifungulia katika kituo cha afya?* *0.* No 1. Yes

Give reason for your answer *Fafanua*…………………..………………………………………………………………..

27c. If given a choice, which health facility would you use for delivery of your next baby? *Ingekuwa ni chaguo lako, je ungependa kuzalia kituo kipi/ au hospitali ipi na kwanini?*

Name and reason *(Jina na sababu)*………………………………………………………………………………..

………

27d. Will you use the health facility near to you **mentioned in Q 17** for delivery of your next baby? *Je ukijaliwa ujauzito mwingine utatumia kituo cha karibu na wewe kujifungulia? Sababu?*

0. No 1. Yes

Give reason for your answer *Fafanua*……………………..…………………………………………………………

27e. For those who delivered at health facility, will you use the facility you used for the delivery of your last baby? *Kwa wale waliojifungulia hospitali/kituo cha afya katika ujauzito wa mwisho, je ungependa kutumia kituo/hospitali ile ile au utabadili? Sababu?*

0. No 1. Yes

Give reason for your answer *Fafanua*…………………………………………………………………………………

28. Will you attend for **postnatal care** within 2 days after delivery of your next baby? *Je ukijaliwa kupata mtoto mwingine utaweza kwenda kupata huduma za afya ndani ya siku mbili baada ya kujifungua?*

No 1. Yes

Give reason for your answer *Fafanua*……………………..……………………………………………………………..

28b. If given a choice, which health facility would you like to use for postnatal care after delivery of your next baby? *Kama ingekuwa chaguo lako, je ungependa kuhudhuria kliniki ipi baada ya mtoto kuzaliwa iwapo utajaliwa mtoto mwingine?*

Name and reason *(Jina na sababu)*………………………………………………………………………………..

………

28c. Will you use the health facility near to you for postnatal care for your next baby? *Je ungependa kutumia kituo kilicho karibu na kwako kwa huduma ya mama na mtoto baada ya kujifungua iwapo utajaliwa mtoto mwingine?*

0. No 1. Yes

Damian et al, 2020

Give reason for your answer *Fafanua*…………………..…………………………………………………………………

29. In general, will you advice women to deliver at health facilities? *Kwa mtazamo wako, unashauri akina mama katika eneo hili wajifungulie katika kliniki au hospitali? Sababu?*

0. No 1. Yes

Give reason for your answer *Fafanua*………………………………………………………………………………

V. Breastfeeding and contraceptive use after last born delivery *Unyonyeshaji na uzazi wa mpango baada ya kujifungua mtoto wa mwisho*

Sex of the last born baby *Jinsia*……………………………

Date of birth of the baby (dd, mm, yyyy) *Tarehe ya kuzaliwa* ……………………………………………...

Age of the baby in months *Umri wa mtoto kwa miezi*………………………………………………….……..

30. How long after delivery did you start breastfeeding the baby? *Je ulianza kumyonyesha mtoto wa mwisho muda gani baada ya kuzaliwa?* ....hours *masaa*

30.2: Was the baby given anything before starting to breastfeed? *Je mtoto alipewa kitu chochote kabla ya kuanza kunyonya maziwa ya mama?*

0. No 1. Yes (*what?..)*

30.3: Was the baby given colostrum? *Je mtoto alipewa maziwa ya mwanzo au yalikamuliwa chini/ kumwagwa?* 0. No 1. Yes

31. Is the baby still breastfeeding? *Je mwanao wa mwisho bado ananyonya?*

0. No (*if not why?...)*

1. Yes

32. Frequency of breastfeeding per day for those with children below 6 months? *Mtoto ananyonya mara ngapi kwa siku?* ....(correct answer, on demand)

33. When did you introduce the following? *Je mtoto alianza kupewa vifuatavyo na umri gani?*

Maji ya kunywa……………………………………………………………………………*in months*

Maziwa ya ngombe……………………………………………………………….…………………..

Ujimwepesi……………………………………………………………………………………………

Supu…………………………………………….……………………………………………………

Ugalmwepesi…………………………………………………………………………………………

Other foods introduced before 6 months?...

34. Is the child given any other foods apart from breast milk at the moment? List all the foods given *Je, je mtoto anapewa vyakula vingine zaidi ya maziwa ya mama? Orodhesha vyakuala apewavyo mtoto*……………………………………………………………………………………….……………

……

Damian et al, 2020

35. Does the child have any problems? *Je mtoto ana shida yoyote ya kiafya?*...

Unmet need for contraceptives after delivery of last baby

36. Are you planning to have another child? *Unampango wa kuwa na mtoto mwingine?*

0. No (*why not?...)*

1. Yes (*when?/after how long?* ***In years*****...)*

37. Have you resumed sex with your partner since delivery? *Umeshaanza kukutana na mwenzi tangu ujifungue?* 0. No

1. Yes (*when after delivery?...)*

38. Have you started menstruating? *Je umeshaanza kutumika au kuona siku zako?*

0. No

1. Yes (*how long after delivery?..months)*

39. Are you **currently** using any contraceptive method to avoid becoming pregnant? *Je kwa sasa unatumia njia yoyote ya kujikinga ili kuzuia usipate ujauzito?*

0. No --- go to Q 42

1. Yes (*time after delivery when started the method…………………………….)*

40. Type of contraceptive method **currently** used *Je ni njia ipi ya uzazi wa mpango unayotumia kwa sasa? Zungushia zote atakazosema anatumia* ***kwa sasa***

1. Male condoms *mpira/ kondumu ya kiume*
2. Oral contraceptive pills *Vidonge*
3. Injectables (Depo-provera) *Sindano/ Depo*
4. Implant *Vipandikizi*
5. IUCD *Kitanzi*
6. Bilateral tubal ligation (BTL) *Kufunga uzazi wa mwanamke*
7. Vasectomy *Kufunga njia ya uzazi ya mwanaume*
8. Others……..…………………………………………………………….

41. Have you **ever** used any contraceptive method? *Umeshawahi kutumia njia yoyote ya uzazi wa mpango tangu ulipoanza mapenzi? Taja njia zote alizowahi kutumia*

0. No

1. Yes (*Mention methods ever used…………………………….…………………………………*

……

……………………..

42. Why aren't you using any modern contraceptive method? *Kwanini hutumii njia yoyote ya kujikinga na ujauzito?*……………………………………….………………………………………………………

Damian et al, 2020

………

43. Does your partner support use of contraceptives? *Je mwenzi wako anakubali utumiaji wa uzazi wa mpango?* 0. No 1. Yes

VII: Support by family to use health facilities for MNCH services *Msaada, sapoti na ushirikiano wa familia kumuwezesha mama mjamzito kutumia vituo vya afya kwa huduma*

43. Regarding your partner (tick the appropriate answer) *Je mume au mwenzi wako (tiki jibu atalosema);*

	0. No	1.Yes
a. Does he think it is important for a pregnant woman to attend at health facility for antenatal care? *Anaamini ni muhimu kwa mama mjamzito kuhudhuria kliniki?*		
b.Did he allow you to attend for ANC in the last pregnancy? *Alikuruhusu kuhudhuria kliniki katika ujauzito wako wa mwisho?*		
c. Does he believe a woman should deliver at home? *Anaamini ni vizuri mama akijifungulia nyumbani?*		
d. Does he believe a woman should deliver at health facility? *Anaamini ni vizuri mama akijifungulia katika hospitali au kituo cha afya?*		
e. Will he support you to deliver at health facility if you will have another pregnancy? *Atakusaidia na kukupa ushirikiano ili ujifungulie katika kituo cha afya katika ujauzito ujao?*		
How will he support you to deliver at the facility? Permission?, money for transport?, please explain in detail *Elezea mwenzi atakusaidiaje ili uweze kujifungulia katika kituo cha afya*		
f. Je mume/mwenzi anaweza kukuzuia usiende kuzalia katika kituo cha afya?		

44. Regarding your mother in law; *Je mama mkwe wako*

	0. No	1.Yes
a. Is she still alive or around? *Je mama mkwe wako yupo?*		
b. Do you live with your mother-in-law at your home? *Je mnaishi nyumba moja na mama mkwe?*		
c. Does she think or believe it is important for a pregnant woman to attend for antenatal care at the health facility? *Anaamini ni muhimu kwa mama mjazito kuhudhuria kliniki akiwa mjamzito?*		
d. Does she believe a woman should deliver at home? *Anaamini ni vizuri mama akijifungulia nyumbani?*		
d. Does she believe a woman should deliver at health facility? *Anaamini ni vizuri mama akijifungulia katika hospitali au kituo cha afya?*		
e. *Je mama mkwe anaweza kukuzuia au kukukatalia usiende kuzalia katika kituo cha*		

Damian et al, 2020

afya?		
f. Will she support you to deliver at health facility if you will have another pregnancy? *Je mama mkwe wako ni mtu anayeweza kukusaidia na kukupa ushirikiano ili ujifungulie katika kituo cha afya katika ujauzito ujao?*<br><br>*Kama ndiyo, atakuasaidia vipi? Elezea*		

45. Regarding your father in law; *Je baba mkwe wako;*

	0. No	1.Yes
a. Is he still alive or around? *Je baba mkwe wako yuko?*		
b. Do you live with your father-in-law at your home? *Je mnaishi na baba mkwe nyumba moja?*		
c. . Does he think or believe it is important for a pregnant woman to attend for antenatal care at the health facility? *Anaamini ni muhimu kwa mama mjazito kuhudhuria kliniki akiwa mjamzito?*		
d. Does he believe a woman should deliver at home? *Anaamini ni vizuri mama akijifungulia nyumbani?*		
d. Does he believe a woman should deliver at health facility? *Anaamini ni vizuri mama akijifungulia katika hospitali au kituo cha afya?*		
e. *Je baba mkwe anaweza kukuzuia au kukukatalia usiende kuzalia katika kituo cha afya?*		
e. Will he support you to deliver at health facility if you will have another pregnancy? *Je baba mkwe wako ni mtu anayeweza kukusaidia na kukupa ushirikiano ili ujifungulie katika kituo cha afya katika ujauzito ujao?*<br><br>*Kama ndiyo, atakuasaidia vipi? Elezea*		

46. General in your cultural beliefs, how does the community perceive a woman who delivers at the hospital or health facility? *Je kwa ujumla, katika mila na desturi zenu, jamii yenu inamchukuliaje mama aliyejifungulia katika kituo cha ya afya?*

47. Again in your opinion what key things should be done or improved so that women would be able and would like to deliver at health facilities? *Kwa ushauri wako ni nini kifanyike ili akina mama waweze na wapende kujifungulia katika vituo vya afya?*
Upande wa serikali?
Upande wa kliniki zenyewe?

Damian et al. 2020

Upande wa jamii?
Upande wa familia?
______________xx____________xx___________xx___________xx______________xx_____________
We have come to the end of questionnaire. *Mwisho wa mahojiano.* Do you have any questions or any concern concerning the services given during pregnancy, childbirth or during postpartum period which you would like to discuss?

Record key concerns to discuss with health facility or district administration:

Damian et al, 2020
